# Comments on Ricardo F Savaris' Letter to the Editor regarding the publication in the JBSTM-Brazilian Protocol for Sexually Transmitted Infections, 2020: “Pelvic Inflammatory Disease”

**DOI:** 10.1590/0037-8682-0457-2021

**Published:** 2021-11-12

**Authors:** Maria Luiza Bezerra Menezes, Paulo Cesar Giraldo, Iara Moreno Linhares, Neide Aparecida Tosato Boldrini, Mayra Gonçalves Aragon

**Affiliations:** 1 Universidade de Pernambuco, Departamento Materno-Infantil, Recife, PE, Brasil.; 2 Universidade Estadual de Campinas, Departamento de Tocoginecologia, Campinas, SP, Brasil.; 3 Universidade de São Paulo, Disciplina de Ginecologia, Departamento de Obstetrícia e Ginecologia, São Paulo, SP, Brasil.; 4 Universidade Federal do Espírito Santo, Departamento de Ginecologia e Obstetrícia, Vitória, ES, Brasil.; 5 Ministério da Saúde, Secretaria de Vigilância em Saúde, Brasília, DF, Brasil.; 6 Universidade Federal do Espírito Santo, Programa de Pós-Graduação em Doenças Infecciosas, Vitória, ES, Brasil.

**Reply to the letter to the editor**

We would like to thank Dr. Savaris for his relevant comments on our article (https://doi.org/10.1590/0037-8682-0419-2021)^1^. His letter emphasizes three basic points: 1) diagnostic criteria for pelvic inflammatory disease (PID), 2) benefits in the use of gentamicin as a single dose rather than in two or three daily doses, and 3) suitability of antibiotic usage prior to intrauterine device (IUD) removal or even the need for its removal for the treatment of PID.

We would like to emphasize that in all our lectures or written articles on female pelvic infections, we always stress the unique characteristics of the 60% to 70% of cases that are asymptomatic^2^. Therefore, to base diagnosis solely on clinical criteria may be insufficient. If the focus is to prevent sequelae, reliance on clinical criteria would result in a delay in early intervention. The more clinical signs that are present, the greater the diagnostic specificity. However, this also corresponds to a lower diagnostic sensitivity. We will fail to treat many women who may have subsequent serious problems with fertility, chronic pelvic pain, or ectopic pregnancy in a timely manner due to the absence of an early diagnosis. In addition, reliance on Dr. Savaris' suggested criteria may increase the number of false positives, as many cases of urinary tract infection or even adnexal cysts can cause pain in the lower abdomen and vagina. This consequently results in unnecessary aggressive treatments. Both laboratory and diagnostic imaging components are essential, regardless of whether they are labeled “major” or “minor” criteria. Thus, a presumptive early diagnosis must be based on complementary tests to effectively reduce the occurrence of adverse sequelae.

Regarding the use of gentamicin in a single daily dose, as shown in Figure 5 "Pelvic inflammatory disease treatment" in our publication^1^, we emphasize that this is the recommended dosage for in-hospital treatment, as it is at least as effective, or perhaps even more effective, than a fractionated dose. It is of lower cost, requires less intervention, and has lower nephrotoxicity than multiple daily dose regimens. It should be noted that aminoglycosides have historically been administered in multiple daily doses (usually 2-4 times/day). Since toxic effects depend more on the duration of therapeutic levels than on maximum drug levels, and pharmacological efficacy depends more on concentration than on time, frequent administrations should be avoided^3^ ^,^ ^4^. However, we caution that single daily regimens are not optimal for all patients. They should not be used in patients with a creatinine clearance above 25 mL/minute, preadolescents, elderly, pregnant or obese women, or in those with burns, ascites, or certain serious infections (such as meningitis, osteomyelitis, skin infection, infection of skin structures, and enterococcal endocarditis)^5^.

The last point raised concerns regarding the removal of an IUD in women with PID. The biological plausibility and similarity with other infections associated with prostheses and orthotics (orthopedic, cardiac, dental, ophthalmic, etc.) support the main recommendation to remove the foreign body. There is always the possibility that their presence facilitates biofilm formation, which reduces or prevents an adequate treatment response or predisposes patients to relapses. We reiterate, as stated in the article, that IUD removal is not necessary in mild and moderate cases of PID, based on European and UK studies^6^ ^,^ ^7^, and the WHO´s medical eligibility criteria for the use of contraceptive methods^8^. However, we emphasize that in severe cases, it is essential to remove this foreign body to optimize treatment. Therefore, we follow the recommendation to not remove the IUD during treatment of PID unless the patient requests its removal or when there is no clinical improvement after 72 hours of adequate antibiotic treatment^9^ ^,^ ^10^. In cases of severe PID, removal of the IUD is recommended after initiation of an antibiotic regimen (level of recommendation I-B)^9^.

Finally, we wish to clarify that our article was prepared based on the Clinical Protocol and Therapeutic Guidelines for Comprehensive Care for People with Sexually Transmitted Infections (PCDT-IST), published by the Ministry of Health of Brazil^11^. Clinical Protocol and Therapeutic Guidelines (PCDT) are documents that establish the criteria for diagnosing infections, diseases, or health problems. They further recommend treatment with medications and other products, list appropriate dosages, and suggest protocols for clinical control mechanisms and for the monitoring and verification of therapeutic results by health professionals and managers of the Brazilian National Health System. The PCDT criteria are based on scientific evidence and criteria of efficacy, safety, and cost-effectiveness of the recommended technologies. They are periodically reviewed every two years. PCDT documents undergo analysis and approval by Conitec (National Commission for the Incorporation of Technologies in the Brazilian National Health System), created by Brazilian Law nº 12.401, of April 28, 2011, which provides for therapeutic care and the incorporation of health technology within the scope of the Brazilian National Health System^12^. The points discussed here may be useful for revisions in the next PCDT-IST update. 
